# Impact of Menstrual cycle-based Periodized training on Aerobic performance, a Clinical Trial study protocol—the IMPACT study

**DOI:** 10.1186/s13063-024-07921-4

**Published:** 2024-01-29

**Authors:** Linda Ekenros, Philip von Rosen, Jessica Norrbom, Hans-Christer Holmberg, Carl Johan Sundberg, Cecilia Fridén, Angelica Lindén Hirschberg

**Affiliations:** 1 Department of Neurobiology, Care Sciences and Society, Division of Physiotherapy, Karolinska Institutet, Alfred Nobels Allé 23, SE-141 83 Huddinge, Sweden; 2 Department of Physiology and Pharmacology, Molecular Exercise Physiology, Karolinska Institutet, Stockholm, 171 77 Sweden; 3 Department of Health Sciences, Lulea University of Technology, 971 87 Lulea, Sweden; 4 Department of Women’s and Children’s Health, Karolinska Institutet, 171 76 Stockholm, Sweden; 5 Department of Healthcare and Welfare, Malardalens University, 721 23, Vasteras, Sweden; 6 Department of Gynecology and Reproductive Medicine, Karolinska University Hospital, 171 76 Stockholm, Sweden

**Keywords:** Menstrual cycle, Female sex hormones, Periodized training, Follicular phase-based training, Luteal phase-based training, Female athlete, Aerobic performance

## Abstract

**Background:**

The menstrual cycle and its impact on training and performance are of growing interest. However, evidence is lacking whether periodized exercise based on the menstrual cycle is beneficial. The primary purpose of this proposed randomized, controlled trial, the IMPACT study, is to evaluate the effect of exercise periodization during different phases of the menstrual cycle, i.e., comparing follicular phase-based and luteal phase-based training with regular training during the menstrual cycle on physical performance in well-trained women.

**Methods:**

Healthy, well-trained, eumenorrheic women between 18 and 35 years (*n* = 120) will be recruited and first assessed for physical performance during a run-in menstrual cycle at different cycle phases and then randomized to three different interventions: follicular phase-based training, luteal phase-based training, or regular training during three menstrual cycles. The training intervention will consist of high-intensity spinning classes followed by strength training. The menstrual cycle phases will be determined by serum hormone analysis throughout the intervention period. Assessment of aerobic performance (primary outcome) and muscle strength, body composition, and blood markers will be performed at baseline and at the end of the intervention.

**Discussion:**

With a robust methodology, this study has the potential to provide evidence of the differential effects of exercise periodization during different phases of the menstrual cycle in female athletes.

**Trial registration:**

ClinicalTrials.gov NCT05697263. Registered on 25 January 2023

## Key message

• The menstrual cycle has the potential to affect sporting performance in female athletes. Female athletes do perceive the impact of the menstrual cycle on physical performance.

• This randomized, controlled trial will provide evidence of any differential effects of exercise periodization during different phases of the menstrual cycle in female athletes.

• The results of this study might lead to new training recommendations given to exercising women.

## Administrative information

Note: This protocol is written with the support and guidance of the SPIRIT 2013 and the SPIRIT-outcomes 2022 items [1]. The numbers in curly brackets in this protocol refer to SPIRIT checklist item numbers (see http://www.equator-network.org/reporting-guidelines/spirit-2013-statement-defining-standard-protocol-items-for-clinical-trials/).

**Table:** 

Title {1}	Impact of menstrual cycle-based periodized training on aerobic capacity, a clinical trial study protocol—the IMPACT study
Trial registration {2a, 2b}	ClinicalTrials.gov: NCT05697263. Registered on 25 Jan 2023
Protocol version {3}	Version 1.2 issue date 5 January
Funding {4}	The Swedish Olympic Committee Oura Health Oy Folksam Research Foundation
Author details {5a}	Linda Ekenros*^1^, Philip von Rosen^1^, Jessica Norrbom^2^, Hans-Christer Holmberg^2,3^, Carl Johan Sundberg^2^, Cecilia Fridén**^1,4,5^, Angelica Lindén Hirschberg**^4,6^ 1: Department of Neurobiology, Care Sciences and Society. Division of Physiotherapy, Karolinska Institutet, Alfred Nobels Allé 23, SE-141 83 Huddinge, Sweden 2: Department of Physiology and Pharmacology, Molecular Exercise Physiology, Karolinska Institutet, 171 77 Stockholm, Sweden 3: Department of Health Sciences, Lulea University of Technology, 971 87 Lulea, Sweden 4: Department of Women’s and Children’s Health, Karolinska Institutet, 171 76 Stockholm, Sweden 5: Department of Healthcare and Welfare, Malardalens University, 721 23 Vasteras, Sweden 6: Department of Gynecology and Reproductive Medicine, Karolinska University Hospital, 171 76 Stockholm, Sweden * corresponding author ** shared last authorship
Name and contact information for the trial sponsor {5b}	Investigator-initiated trial: Angelica Lindén Hirschberg (principal investigator), email: angelica.hirschberg.linden@ki.se
Role of sponsor {5c}	This study is initiated by the authors (the investigators), and the sponsor is thereby the institution where the primary investigator is appointed: Department of Gynecology and Reproductive Medicine, Karolinska University Hospital, Stockholm, Sweden. The sponsor holds the indemnity insurance and legal liability. The funders play no role in the study design; the collection, analysis, and interpretation of the data; and the writing of the manuscript.

## Introduction

### Background and rationale {6a}

The menstrual cycle has the potential to affect sporting performance in female athletes. There is a growing interest of how exercise and performance can be optimized according to the menstrual cycle [2]. However, the effect of the hormonal variation on physical performance, as well as the impact of menstrual cycle-related symptoms, is only partly understood [3, 4].

Serum levels of the female sex hormones estradiol (E2) and progesterone (P4) vary greatly during the three phases of the menstrual cycle, i.e., the follicular phase (the first day of menstruation to ovulation), the ovulatory phase (at mid-cycle, 1–2 days), and the luteal phase (from ovulation to the next menstrual bleeding). E2 and P4 have receptors in most tissues of the human body and have important roles beyond the reproductive system [5]. Receptors for E2 (ERα and ERβ) have been detected in human skeletal muscle tissue [6, 7]. We have shown a substantial variation of receptor expression for ERs and for the progesterone receptor (PR) in the skeletal muscle throughout the menstrual cycle [8], which might influence neuromuscular performance and the effect of training during the menstrual cycle [8, 9].

It has been hypothesized that periodization of training in relation to the menstrual cycle can impact physical performance. Intensified strength training during the follicular phase has been proposed to improve muscle strength more than the same frequency of training during the luteal phase [10–13]. However, in a recent umbrella review with meta-analysis, the authors suggest that due to the methodological shortcomings in the reviewed articles, it would be highly premature to conclude that short-term fluctuations in ovarian hormones and their receptor levels in relevant target tissues noticeably influence acute exercise performance or longer-term adaptations to resistance training [3]. The authors call for evidence-based approaches for training recommendations for female athletes, based on research utilizing accurate and reliable menstrual cycle phase detection methods [3].

Menstrual cycle-related symptoms may affect physical exercise and performance. Menstrual-related pain, i.e., primary dysmenorrhea, is reported to affect most female athletes which often leads to absence from planned training [14, 15]. Furthermore, premenstrual symptoms (PMS) are experienced by most fertile-aged women and can be characterized by both physical symptoms (e.g., bloating, breast tenderness, headache) and psychological symptoms (e.g., depression, irritability, fatigue) occurring from the mid-luteal phase to the first days of menstrual bleeding [16, 17]. These symptoms have been reported to negatively influence athletic performance and well-being in female athletes [4, 14, 15].

### Objective {7}

The main objective of this planned trial, Impact of Menstrual cycle-based Periodized training on Aerobic performance: a Clinical Trial, "the IMPACT study", is to evaluate the effect of exercise periodization during different phases of the menstrual cycle, i.e., comparing follicular phase-based and luteal phase-based training with regular training throughout the menstrual cycle on physical performance in well-trained women.

Our primary hypothesis is that follicular phase-based training is superior to both luteal phase-based training and regular training throughout the menstrual cycle as it improves aerobic performance and muscle strength at higher extent.

The secondary hypothesis is that a variation in muscle morphology, e.g., gene expression, metabolic enzymes, markers of muscle protein synthesis, will be shown as a training effect, depending on the intervention group.

### Trial design {8}

**Fig. 1:**
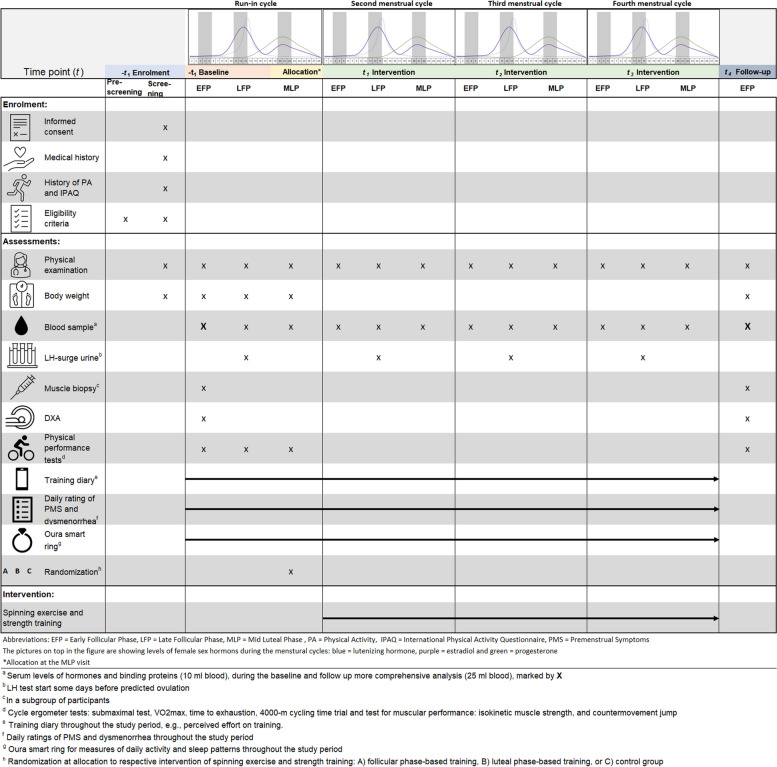
Illustration of the study design with enrollment, assessments, and interventions. The early follicular phase (EFP) is set to cycle days (CD) 3–5, the late follicular phase (LFP) to CD 11–13, and the mid-luteal phase (MLP) to CD 20–22. The assessment of physical performance test at the LFP and MLP in the first menstrual cycle (run-in cycle) is not a part of the intervention

The IMPACT study is a randomized, controlled trial, in which 120 exercising women will be allocated to three parallel groups, i.e., follicular phase-based training, luteal phase-based training, or regular training during three menstrual cycles. The IMPACT study will be preceded by a run-in cycle, see Fig. 1 for the study design. For reports, the trial will use the CONSORT 2010 explanation and elaboration: updated guidelines for reporting parallel group randomized trials [18] and the REPORT Trial guide for effective and transparent research reporting [19].

## Methods: participants, intervention, and outcomes

### Study setting {9}

The study will be carried out at research units at Karolinska Institutet (KI) and Karolinska University Hospital, Stockholm, Sweden. The training intervention will be carried out at specific healthcare centers in the Stockholm area. The recruitment is set to start in August 2023, and the study will run until the end of 2025.

### Eligibility criteria {10}

Healthy women with a regular aerobic training background will be assessed for eligibility in the study. The inclusion and exclusion criteria are listed in Table 1.

**Table 1:** Criteria for eligibility

Inclusion criteria	Exclusion criteria
Females aged 18–35 years	Chronic disease
Regular menstruation (26–32 days interval)	Neurological disorder
Having a BMI within the interval of 19–26 kg/m^2^	Musculoskeletal injury in the last 6 months
Exercising ≤ three times/week the last 6 months	Irregular menstruation
Being able to fulfill the intervention period	Pregnancy or lactation in the last 6 months
	Use of hormonal contraceptives the last 3 months
	Use of regular medication for the last 3 months

*BMI* body mass index

### Who will take informed consent? {26a}

Potential participants will be given oral and written information about the aims of the study and its procedure. If eligible according to the inclusion and exclusion criteria, they will be asked to give their written consent to participate. The project coordinator will take the informed consent and scan it into the data storing system: Research Electronic Data Capture (REDCap®)

### Additional consent provision for collection and use of participant data and biological specimens {26b}

On the consent form, it is stated in a separate paragraph that participants give consent to the collection of blood plasma and biopsy specimens to be stored in a research biobank. The biobank is under the legal supervision of the Swedish Data Protection Regulation. The consent to collection of biopsy specimens will be given of a subgroup of participants.

Upon enrollment, a comprehensive anamnesis of each participant will be performed. This aims to review gynecological history, general health, medication, historical and recent training habits, sleeping, and nutritional status. Physical training patterns in the past and present will be evaluated using the International Physical Activity Questionnaire (IPAQ) [20].

#### Run-in cycle

The study starts with a run-in cycle including assessments at the early follicular phase used as a baseline for the IMPACT study (Fig. 1). The purpose of the run-in cycle is to compare physical performance in different phases of the menstrual cycle without periodization and to assess women’s individual cycle.

During the run-in cycle, the participants will be assessed for the outcome’s measures at three phases during the first menstrual cycle (Fig. 1):

- *Early follicular phase (EFP)*, cycle days (CD) 3–5 (low levels of E2 and P-4)
- * Late follicular phase (LFP)*, CD 11–13 (high levels of E2)
- *Mid luteal phase (MLP)*, CD 20–22 (high levels of E2 and P-4)

Cycle day for examination will be adapted to the participant’s cycle length. The first visit during the run-in cycle is the baseline in the IMPACT study.

Prior to the test protocol, a 10-min warming up on a cycle ergometer will be performed. The test protocol includes several tests of physical performance (see the “Outcomes” section) which will take approximately 75 min/participant, with standardized rest between every test. Nutritional recommendations will be provided to the participants to standardize the diet 24 h prior to the day of testing. In addition, participants will be required to keep an unchanged diet throughout the study period.

#### Run-in cycle 1st visit/baseline

- A fasting blood sample of 25 ml and a urine sample will be collected in the morning between 08:00 and 10:00 AM before breakfast and after refraining from alcohol, caffeine consumption, and any intense physical activity or sports practice 24 h prior to the testing day for analysis of hormones and binding proteins.
- A light breakfast will be served.
- Physical performance tests including aerobic performance and aerobic capacity, peak power output, isokinetic muscle strength, and jumping performance will be performed.
- Body composition will be measured by dual-energy X-ray absorptiometry (DXA).
- Subjective rating of menstrual cycle-related symptoms, e.g., dysmenorrhea and PMS, will be performed every day from baseline and during the entire study period.
- Registration of daily physical activity, sleeping pattern, heart rate, and heart rate variability will be monitored by the Oura smart ring (Oura Health Ltd., Oulu, Finland).
- Muscle biopsy will be obtained under local anesthesia from the m. vastus lateralis in a subgroup of participants (*n* = 45). The muscle biopsies will be collected at EFP, the day after the physical assessments.

#### Run-in cycle 2nd visit

*•* A non-fasting blood sample of 10 ml will be collected for the determination of the cycle phase.

*•* Physical performance tests include aerobic performance, aerobic capacity, peak power output, isokinetic muscle strength, and jumping performance.

*•* For the detection of ovulation, the participants will use urinary sticks (Ovustix, Clearplan, Unipath Limited, Bedford, UK) measuring the luteinizing hormone (LH) surge.

#### Run-in cycle 3rd visit

*•* A non-fasting blood sample of 10 ml will be collected for the determination of the cycle phase.

*•* Physical performance tests include aerobic performance, aerobic capacity, peak power output, isokinetic muscle strength and endurance, and jumping performance.

*•* Randomization to one of the two intervention groups or control group.

### Intervention

#### Explanation for the choice of comparators {6b}

In this randomized controlled trial, we have chosen to include well-trained females and will randomly assign them to the regimen of extended sessions of exercise during the follicular phase or during the luteal phase in the menstrual cycle and the control group of regular exercise throughout the menstrual cycle. All participants irrespective of intervention group or control will over the total intervention period obtain the same numbers of training sessions.

#### Intervention description {11a}

**Fig. 2:**
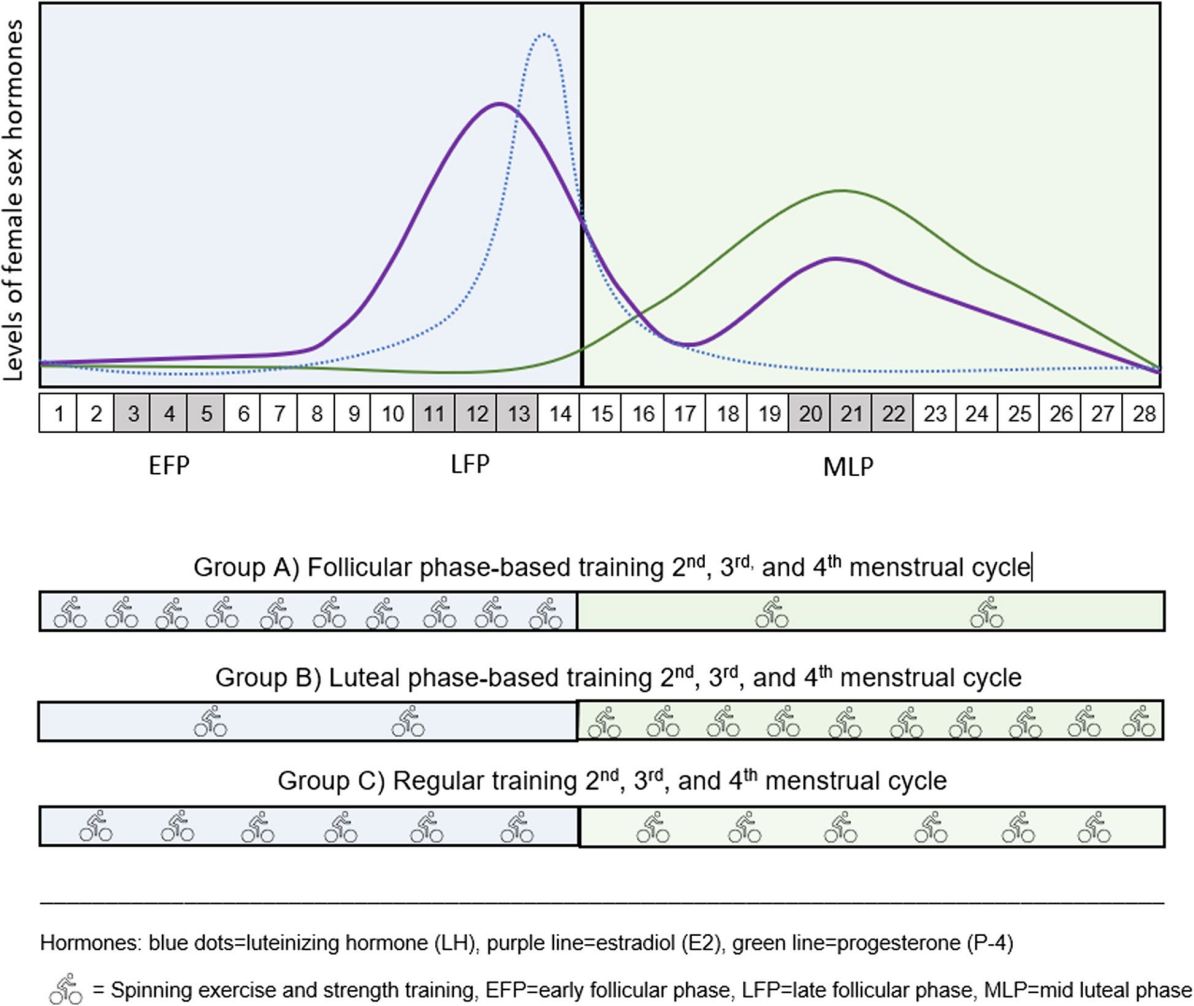
The disposing of spinning exercise and strength training in the two intervention groups (**A**, **B**) and the control group (**C**) in an average 28-day menstrual cycle

The participants will be randomized to different training scheduling of spinning and strength training during the following three menstrual cycles (Fig. 2).

- *Group A*: follicular phase-based training (spinning and strength training) five sessions per week during the follicular phase (approx. 14 days) and thereafter once a week during the luteal phase (approx. 14 days)
- *Group B*: luteal phase-based training (spinning and strength training) once a week during the follicular phase (approx. 14 days) and thereafter five sessions per week during the luteal phase (approx. 14 days)
- *Group C*: regular training (spinning and strength training) three times a week throughout the study period (control group)

In total, there will be 12 sessions of exercise per menstrual cycle during 3 consecutive cycles for all 3 groups.

At the end of the run-in cycle, randomization will be performed, and the participants will start the intervention immediately, at the following first day of menstruation. The intervention lasts for 3 menstrual cycles. The training program during the intervention consists of spinning exercise and muscular strength training, at the training centers in the Stockholm area, Sweden. The spinning exercises consist of 55 min of intermittent, high-intensity training (75–90 percent of maximal heart rate) with recovery periods in between. The heart rate during the spinning will be followed and registered on each occasion during the study period. Additionally, during at least 1 session per week, the total work in Joule will be monitored, utilizing a Wattbike during the exercise, and participants will be responsible for reporting these values in the REDCap® system. The strength training program consists of five exercises for the lower body (squats, lunges, hip thrust, and for core muscles back extension and isometric trunk, e.g., “plank”). The load and the number of repetitions of the strength training will be based on the participants’ fitness level and tested out during the run-in cycle. The range of repetition will be within 8–12 repetitions, and the number of sets will range within two to four sets.

During every training session, the participants will register their perceived exertion as well as the rating of perceived motivation prior to exercise in the REDCap®. To confirm the specific phase of the menstrual cycle, blood samples will be collected according to the EFP, LFP, and MLP (Fig. 1).

#### Criteria for discontinuing or modifying allocated intervention {11b}

The participants will have the full right to discontinue the intervention and their study participation at any time, without having to explain the reason. In case of temporary illness (e.g., cold or flu) that causes the inability to exercise, the intervention period will be extended. The decision about the extension of the intervention period is made in each individual case by the project management group. If a participant develops symptoms and signs of mental and/or physical illness during the assessment of physical tests or during the intervention, the formal stopping rules will apply. Modification of allocated intervention will not be made.

#### Strategies to improve adherence to intervention {11c}

Adherence to this protocol is judged to be high, as it does not consist of very strict guidelines. Strategies to improve adherence to the intervention protocol include reminders by a daily text message to each participant. To evaluate the amount of exercise completed by every participant, each training session will be documented (attendance, total time of exercise, average heart rate, perceived exhaustion, and motivation prior to exercise).

#### Relevant concomitant care permitted or prohibited during the trial {11d}

The use of regular medication during the study is not permitted, but temporary use of, e.g., analgetic due to headache could be allowed based on individual judgment. Other treatments will not be allowed.

During the intervention period, the participants are not allowed to carry out heavy or intense training (above scale number 12 on the Borg RPE scale) apart from the training schedule given by allocation. The participants are free to take part in light daily activities, like commuting to work by bike or walking.

#### Provisions for post-trial care {30}

There is no anticipated harm and compensation for trial participation. This study focuses on health and wellness, and all participants will be given the same amount of training throughout the trial. There is no provision for post-trial care.

### Outcomes {12}

#### The primary and secondary outcomes assessed at baseline and at follow-up at week 17

The outcome measures are listed in Table 2. The primary outcome measure is the aerobic performance of cycling time to exhaustion (TTE) during an individually incremental workload on a cycle ergometer. Secondary outcome measures are aerobic capacity of gas exchange (VO_2max_) on a cycle ergometer, 4000-m self-paced cycling time trial (TT), muscle strength (isokinetic peak torque), jumping performance (countermovement jump), ratings of menstrual-related symptoms, body composition, and muscle tissue variables (in a sub-sample of participants).

**Table 2:** Outcome measures

Outcome	Instrument	Domains	Unit
*Laboratory test*			
Time to exhaustion (TTE)*	Incremental cycle ergometer test	Aerobic capacity and exhaustion	Time in min:s
VO_2max_	COSMED, incremental cycle ergometer test	Aerobic capacity, and gas exchange	ml O_2_/kg/min
Submaximal VO_2max_	Åstrands test	Aerobic capacity	Estimated ml O_2_/kg/min
4000-m cycling time trial	4000-m cycle ergometer test	Aerobic capacity	Time in min:s
Isokinetic muscular strength	Biodex at 60°/s	Muscular strength, knee extensors, and knee flexors	Newton meter (Nm) peak torque
Fat mass, fat-free mass, mineral density	Dual-energy X-ray absorptiometry (DXA)	Body composition	Mass and density
Muscle morphology	Weil-Blakesley conchotome, muscle biopsy	Muscle morphology	
Blood markers	Blood sampling	Blood cells, genetics, biochemistry, and hormonal levels	
Capillary blood lactate	Blood sampling	Lactate	mmol/l
*Clinical test*			
Hop function	Countermovement jump	Explosive muscular function	Centimeters (cm)
*Questionnaires*			
Effort perception	Borg scale		Scale number of perceived exhaustion (6–20)
Premenstrual symptoms	Daily Record of Severity of Problems (DRSP)		Scale number of severity (1–6)
Dysmenorrhea	Numerical Pain Rating Scale (NRS)		Scale number of severity (0–10)
*Free-living assessment*			
Numbers of daily steps	Oura smart ring	Physical activity	Numbers (*n*)
Quality of sleep	Oura smart ring	Sleep pattern	

*Primary outcome measure

### Participant timeline {13}

**Fig. 3:**
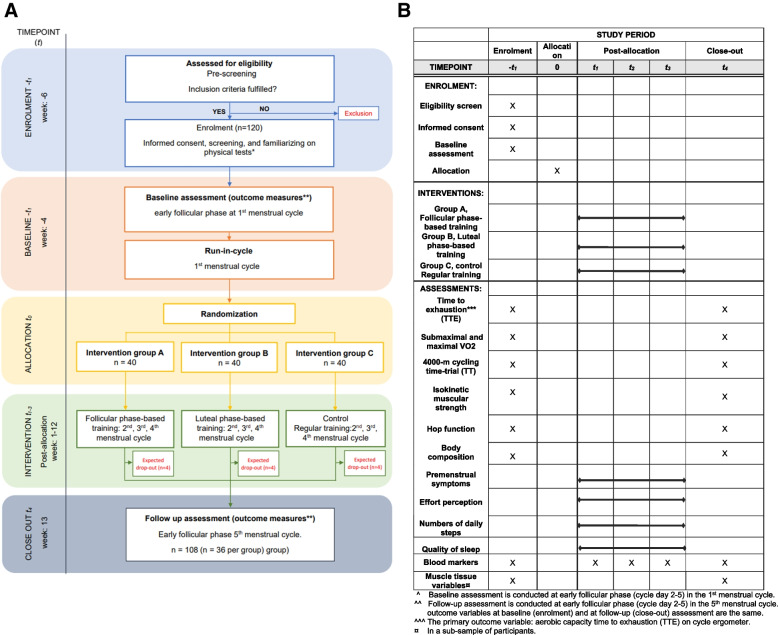
**a** Flowchart and participant timeline. Schedule of enrollment, interventions, and assessments. Time point in the figure: − *t*_1_ = approx. 6 weeks prior to allocation (0), *t*_1_ = weeks 1–4 (2nd menstrual cycle), *t*_2_ = weeks 5–8 (3rd menstrual cycle), *t*_3_ = weeks 9–12 (4th menstrual cycle), and *t*_4_ = week 13. The time points refer to an average 28-day menstrual cycle. * Physical tests = test of aerobic capacity and performance and muscle strenght and jump performance. ** Outcome measures = all outcome measures listed in table 2. **b** Schedule of enrolment, interventions, and assessments according to SPIRIT guidelines 2013. Timepoint in the figure: -t1 = approx. 6 weeks prior to allocation (0), t1 = week 1-4 (2nd menstrual cycle), t2 = week 5-8 (3rd menstrual cycle), t3 = week 9-12 (4th menstrual cycle), t4 = week 13. The timepoints is based on a 28-day menstrual cycle

The time schedule of enrollment, intervention, assessments, and visits of participations is shown in a schematic diagram in Fig. 3a, b.

### Sample size {14}

The power estimation was conducted using the R statistical software (the R Foundation for Statistical Computing, Vienna, Austria), package “SIMR.” To identify an effect size difference of 0.70 SD in TTE (primary outcome), between two of the groups, 33 individuals need to be included in each group (alpha 0.05, power 80%). For the secondary outcomes, an effect size difference of 0.60 SD is assumed. This results in that 36 individuals should be included in each group. Assuming dropouts (*n* = 12), 40 individuals will be included in each group, which will give an allocation ratio of 1:1. No power calculation is performed for the exploratory outcomes such as muscle tissue variables.

### Recruitment {15}

The participants will be recruited via advertisement at the Karolinska Institutets (KIs)s website for research subjects, via a student’s platform at KI medical education and at the Swedish School of Sports and Health Sciences. Recruitment announcements will be posted online and at sports and healthcare universities in the Stockholm area, Sweden.

### Assignments of interventions: allocation

#### Sequence generation {16a}

A computer-generated random number list (allocation table), aiming to randomly order the participants to the three study groups, will be prepared by a statistician with no involvement in the trial.

#### Concealment mechanism {16b}

Allocation is not concealed and will be revealed to both the participant and the researcher upon randomization.

#### Implementation {16c}

The allocation sequence is generated by a statistician with no involvement in the trial. The REDCap® randomization module will be utilized to implement and upload the allocation table in the system as well as allocate the participant to one of the three groups. The study group will be revealed at the same time for both participant and researcher.

### Assignments of interventions: blinding

#### Who will be blinded {17a}

Blinding of intervention for the participants will not be possible for obvious reasons. Furthermore, the study coordinator will give individual instructions about the specific training program according to group distribution and individual cycle length. However, analysis of study outcomes will be performed by a researcher blinded to group allocation.

### Data collection and management

#### Plans for assessment and collection of outcomes {18a}

##### Settings

The physical performance tests will be performed at the u-MOVE laboratory for human movement and activity at the Karolinska University Hospital, Stockholm, Sweden. Prior to the data collection at baseline, the participants will visit the laboratory for familiarization tests and to determine performance values.

##### Aerobic capacity and aerobic performance on cycle ergometer test

A submaximal cycle ergometer test described by Åstrand [21] will be performed followed by a progressive test of maximal aerobic capacity (VO_2max_). The protocol for VO_2max_ will be performed by incremental cycling on an electric braked cycle ergometer (Monark 928 G3). The participants will start at 125–150 W (depending on pretest results) and increase 10 W every 20 s until exhaustion, and the cadence drops below 30 revolution per minute (RPM). The primary outcome measure (TTE) will be registered on a separate timer, from the start of the cycling protocol to the point of exhaustion when the test stops. Oxygen uptake and carbon dioxide elimination will be measured by the breath-by-breath method (BxB mode) and together with workload and heart rate will be recorded continuously throughout the test. A portable metabolic system COSMED (K5) will be utilized to measure oxygen consumption (VO_2_) and carbon dioxide production (VCO_2_). The COSMED K5 in BxB mode is considered a valid system for the measurement of ventilation (VE), VO_2_, and VCO_2_ for a wide range of metabolic rates [22]. Rating of perceived exertion (RPE) will be monitored using the Borg RPE scale [23]. The variable for aerobic capacity for gas exchange is measured by ml 0_2_/kg/min.

##### 4000-m self-paced cycling time trial (TT)

The 4000-m self-paced cycling TT [24] will be performed on a cycle ergometer (Monark 928 G3). The participants will start with 3 min cycling at 125 W and then further 3 min at 70% of VO_2peak_. After this 6-min warm-up, the participants will be given a 5-min rest to prepare for the subsequent time trail. The participants will be given the instruction to complete the distance of 4000 m in as short time as possible. Verbal feedback on the distance covered every 400 m will be given, and the heart rate will be recorded at those time points. The participants will be allowed to adjust the gear to a preferred cadence during the trial. A capillary blood sample will be obtained and assessed for lactate (La) concentration immediately after completing the test and at 7 min posttest.

##### Isokinetic muscular strength

The maximal isokinetic strength of the knee extensor muscles and the knee flexor muscles will be assessed by five consecutive maximal isokinetic voluntary contractions at the velocity of 60° per second. Peak torque (Nm) will be used in the analysis. The isokinetic measures will be performed in the Biodex Device®. The Biodex has been tested with acceptable mechanical reliability and validity [25].

##### Countermovement jump

For jump performance, the countermovement jump (CMJ) [26] will be performed, to assess maximal vertical jumping height. A computerized system (MuscleLab, Ergotest Technology) will measure the flight time which will be converted into jump height in centimeters (cm). Three maximal jump height (cm) will be recorded. Between jumps, a break of 30 s was provided. The best trial will be included in further analysis.

##### Ratings of menstrual cycle-related symptoms

Prospective daily ratings of premenstrual symptoms will be performed with the Daily Record of Severity of Problems (DRSP) form using the REDCap® application with notification of daily surveys to the participants. The DRSP form consists of 21 separate items of premenstrual symptoms (physical and psychological) as well as impairment in functioning caused by the symptoms. Ratings will be made on a 6-point severity scale. The DRSP has been evaluated for reliability and validity [27] and used as diagnostic criteria in research studies [28]. The participants will also be asked to daily rate their perceived symptoms of dysmenorrhea, using the REDCap® application. A numeric rating scale (NRS) will serve as a subjective measure of pain intensity for dysmenorrhea [29].

##### Body composition

For the assessment of whole-body composition, the standard method by DXA will be used. The DXA measurements will be performed by using Fuji Nordic, FDX Visionary equipment. DXA offers an assessment of fat mass, fat-free mass, and bone mineral density and is considered safe for the participant (low dose of X-ray), valid, and reliable [30].

##### Muscle biopsy

In a subset of participants (*n* = 45), resting muscle biopsy specimens are obtained at baseline and at follow-up under local anesthesia from the distal portion of the m. vastus lateralis of the quadriceps femoris muscle of one leg using a Weil-Blakesley conchotome (AB Wisex, Mölndal, Sweden) under standardized medical supervision. In the specimen, analyses of fiber composition, relative fiber area, and number of capillaries for each fiber group are performed. The concentration of hormone receptors, metabolic enzymes, markers of muscle protein synthesis, and markers of atrophy will be determined.

##### Tracking of daily activity and sleep patterns

The Oura smart ring (Ōura Health Ltd, Oulu, Finland) will be used throughout the study to monitor physical activity and sleeping patterns. The smart ring has a built-in photoplethysmography sensor, an inertial measurement unit for heart rate, and a 50-Hz triaxial accelerometer for physical activity tracking. The validity of the Oura smart ring has shown promising results for resting heart rate, for sleep duration [31], and for every day, free-living activity.

##### Psychological measures

Motivation and self-perception of performance level will be monitored in connection to every exercise session utilizing a VAS scale (0–10), where 0 indicates the lowest level and 10 the highest level.

##### Laboratory analyses and cycle phase determination

Follicle-stimulating hormone (FSH), luteinizing hormone (LH), and sex hormone-binding globulin (SHBG) will be analyzed by electrochemiluminescence immunoassay at the Department of Clinical Chemistry, Karolinska University Hospital. Serum levels of E2, P4, and testosterone will be determined by liquid chromatography-tandem mass spectrometry (LC-MS/MS) [32]. The free androgen index is calculated based on testosterone nmol/L divided by SHBG nmol/L × 100.

Serum hormone analyses are used to determine the cycle phase. We define the early follicular phase (CD 3–5) as low levels of FSH, LH, E2, and P4; late follicular phase (CD 11–13) as LH levels higher than FSH, high levels of E2, and low levels of P4; mid-luteal phase (CD 20–22) as low levels of FSH and LH and high levels of both E2 and P4 in an ideal MC of 28 days or adapted to an individual MC length.

#### Plans to promote participant retention and complete follow-up {18b}

The participants will, pre-entering the study, receive extensive information about the study and the requirements during the trial. Questionnaires during the study period are conveniently completed by online survey in the REDCap®.

#### Data management {19}

Data will be automatically (e.g., for surveys) or manually (e.g., for results on the physical performance test, serum analysis) entered into the REDCap® system.

#### Confidentiality {27}

Confidentiality of the participants’ data will always be guaranteed in accordance with the General Data Protection Regulation (GDPR). All data will be pseudo-anonymized and de-identified participants’ data obtained in the trial will be kept under the participants’ number. The IMPACT study will be conducted in accordance with the Code of Ethics of the World Medical Association (Declaration of Helsinki). The de-identified data will be used in the data management and only the investigators will be able to access.

#### Plans for collection, laboratory evaluation, and storage of biological specimens for genetic or molecular analysis in this trial/future use {33}

Blood will be drawn from the trial participants on 13 occasions, with a total volume of 160 ml. At baseline assessment (week 1) and at follow-up (week 17), 25 ml blood will be drawn, and at the remaining 11 occasions, an amount of 10 ml blood will be drawn on each occasion (Fig. 1).

Muscle biopsies from the m. vastus medialis will be collected at baseline and follow-up (before and after intervention) in a sub-sample of participants. At each collection, two samples will be taken.

Blood samples and muscle tissue will be stored in the research biobank at − 80 °C until the trial is completed, and all planned analyses are performed. Any additional studies on the collected biological material will need new approval by the Committee of Ethics and approval according to the Swedish Data Protection Regulation and the Swedish Data Protection Law. Trial participants can without any consequences decline to donate blood and muscle tissue for the biobank for future unspecified research. Participants can at any time request to have their biological material destroyed, with such request, the biological material will immediately undergo destruction.

## Statistical methods

### Statistical methods for primary and secondary outcomes {20a}

Results for the parametric variables will be expressed by mean (SD) and non-parametric variables by median (interquartile range) or frequency (percent). Analyses of physical performance variables (described in section outcomes) between different cycle phases (EFP, LFP, MLP) within the run-in cycle will be performed using linear mixed models. The randomization process will lead to balanced groups and no systematic differences in unknown and measured characteristics of the three groups. Change in the respective outcome measure (described in section outcomes) from baseline to follow-up will be assessed.

Linear mixed models will be used to evaluate the effect of exercise periodization on aerobic fitness tests and to explore the effect of exercise periodization on the secondary outcome variables, with the group included as a factor variable. These analyses will be performed using an intention-to-treat approach. Multiple imputations are not needed as linear mixed models handle missing data. The level of significance will be set at *p* < 0.05. All analyses will be performed using SPSS V.23.0 (IBM).

### Interim analyses {21b}

No interim analyses will be performed.

### Methods for additional analyses (e.g., subgroup analyses) {20b}

In per-protocol analyses, we will analyze the effect of exercise periodization on primary and secondary outcomes for the individuals who have completed the intervention protocol.

### Plans to give access to the full protocol, participant-level data, and statistical code {31c}

Our intention is to grant access to the full protocol and group-level data upon reasonable request and for the purpose of increasing scientific knowledge in the field.

### Oversight and monitoring

#### Composition of the coordinating center and trial steering committee {5d}

This trial is performed at a single center. The project management group is composed of a project coordinator and test leader (LE), project leader and sponsor with responsibility for project organization (CF), and a principal investigator and sponsor with overall scientific responsibility for the study (ALH). This group will have regular meetings to ensure adherence to the protocol, the quality of data collection, and safety for participants. There is no steering committee or stakeholder involved in this trial.

#### Composition of the data monitoring committee, its role, and reporting structure {21a}

No data monitoring committee has been considered since this trial is considered a low-risk intervention.

#### Adverse events reporting and harms {22}

Adverse events (of any type) are collected during the trial and urgently reported back to the principal investigator and noted in a separate adverse events document. The primary investigator is responsible for handling the adverse event and referring the participants to the relevant department or clinician if needed. Any serious incidents and adverse events (SAE) will be reported immediately to the Swedish Ethical Committee.

#### Frequency and plans for auditing trial conduct {23}

No auditing of an authority is planned for this trial. The intervention of this trial is considered as low risk.

#### Plans for communicating important protocol amendments to relevant parties (e.g., trial participants, ethical committees) {25}

Important protocol modifications made in the study protocol will be communicated to the ethics committees, the trial register, and the journal where the study protocol is published.

#### Dissemination plan {31a}

The results from this proposed study will be presented at scientific conventions and published in peer-reviewed international academic journals. Further, the results will be disseminated to national and international sports federations through stakeholders and media. Study data will be deposited safely in long-term storage at KI, according to the Local Ethics Committee of Stockholm, Sweden.

## Discussion

The impact of the menstrual cycle on training and physical performance is still not elucidated. Although previous studies on periodized training indicate an eventual advantage for follicular-based training [3], the results are not conclusive. Furthermore, the role of menstrual cycle-related symptoms for training and performance has not been fully explored. A recent study suggested that motivation to exercise is of superior significance rather than variation in hormone levels during the menstrual cycle to improve physical performance [4]. As several menstrual cycle-related symptoms such as dysmenorrhea and PMS are common among athletes, these symptoms might affect motivation and the effectiveness of training sessions.

With a robust methodology and sufficient sample size, the IMPACT study has the potential to provide evidence of the effect of exercise periodization during different phases of the menstrual cycle, which could form the basis for recommendations of training to improve physical performance and maintain health in female athletes. The rationale for the study procedures relies on the findings and recommendations from earlier studies [3]. During the IMPACT study, the included women will be followed prospectively for four consecutive menstrual cycles and be continuously examined during the study period. Besides the main outcome of aerobic capacity, a broad range of comprehensive examinations of physiological, mental, and behavioral parameters will be collected, which will provide opportunities to thoroughly explore potential variables that impact physical performance and trainability in female athletes.

To confirm the menstrual cycle phase, blood and urine samples will be collected throughout the study period for hormone determination. Additionally, the subjects will rate and report their menstrual symptoms, e.g., PMS and dysmenorrhea on a daily basis. The online tool REDCap® will be utilized to send out messages to the participants requesting daily ratings and reminders in case of missing replies. This will lead to more accurate reporting compared to retrospective ratings.

The training intervention will not be supervised in a controlled environment; however, the women’s physical activity and training sessions will be followed continuously using the Oura smart ring. Moreover, during the intervention, the participant will in at least one training session per week monitor and report their achieved total work (J). Furthermore, they will self-score their motivation and perception in terms of their own effort and perceived exhaustion from every exercise session conducted. This will lead to an increased insight into the process behind trainability in female athletes in relation to the menstrual cycle.

The choice of outcome measures to evaluate physical performance was based on the demands of spinning exercises with aerobic intervals and muscular strengthening in the lower extremities. The primary outcome of TTE is widely used in a laboratory setting to measure aerobic performance and the average power output for TTE has shown to be higher compared to a TT protocol [33]. Self-paced TT is considered a more functional test with high ecological validity [33].

The proposed study may have some limitations. The results of the training intervention can only be generalized to female athletes with a moderate to high level of fitness and not to elite athletes. Due to the nature of the intervention, the participants cannot be blinded. Furthermore, the study coordinator will not be blinded to group allocation. However, the researcher analyzing the data will be blinded to the study intervention group.

## Summary

The IMPACT study aims to investigate the effectiveness of different types of periodized training protocols based on the menstrual cycle. The proposed methodology is robust in accordance with recommendations for menstrual cycle studies. The findings will enable menstrual cycle-related evidence-based training recommendations for female athletes. Furthermore, findings will enhance the knowledge of the impact of eventual menstrual cycle-related symptoms on physical performance and training.

## Trial status

Protocol version 1.2, issue date 5 January 2024. Participant recruitment started in August 2023 and will continue until the target number of participants (*n* = 120) has been enrolled. The expected completion of recruitment is around February 2025.

## Data Availability

The principal investigator and the investigators will have access to the final trial data set.

## Abbreviations

CD: Cycle day
CMJ: Countermovement jump
DRSP: Daily record of severity of problems
DXA: Dual-energy X-ray absorptiometry
EFP: Early follicular phase
E2: Estradiol
FSH: Follicle-stimulating hormone
GDPR: General data protection regulation
IPAQ: International physical activity questionnaire
KI: Karolinska institutet
LC-MS/MS: Liquid chromatography-tandem mass spectrometry
LFP: Late follicular phase
LH: Luteinizing hormone
MLP: Mid-luteal phase
NRS: Numeric rating scale
P4: Progesterone
PMS: Premenstrual symptoms
RCT: Randomized controlled trial
RPE: Perceived exertion
SHBG: Sex hormone-binding globulin
TT: Time trial
TTE: Time to exhaustion
VE: Ventilation
VCO2: Carbon dioxide production
VO_2max_: Maximal aerobic capacity
